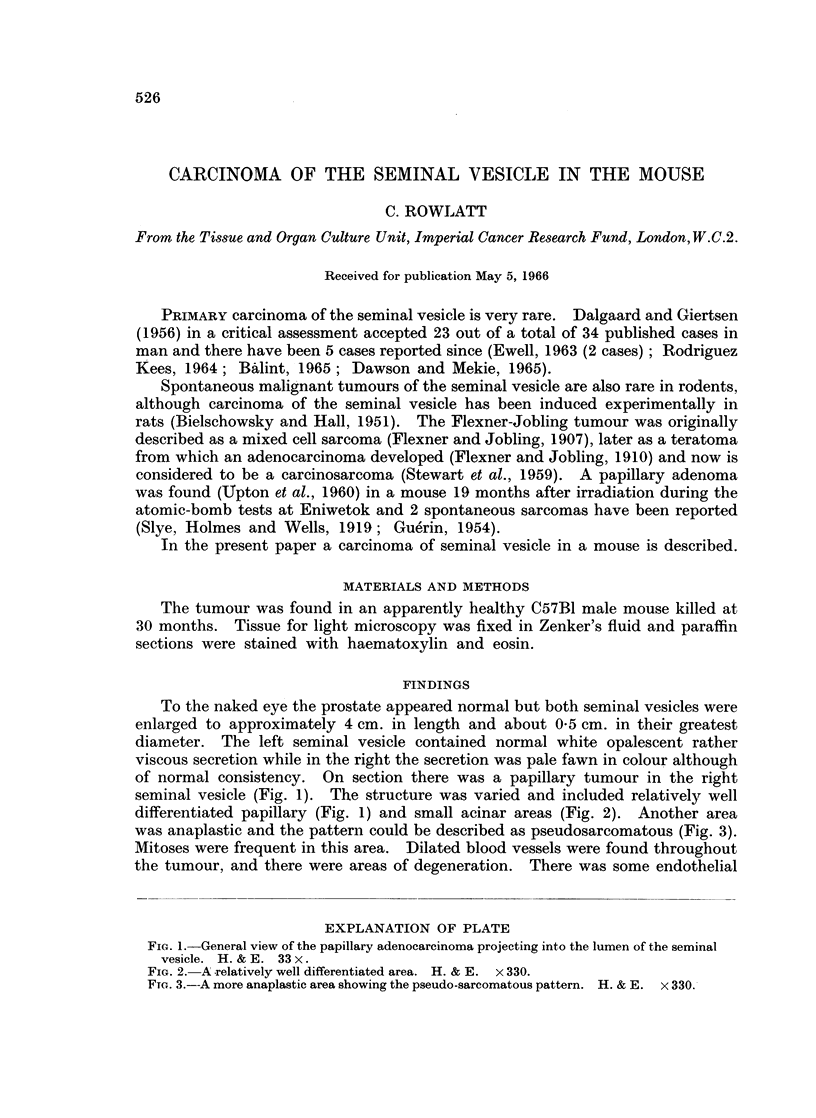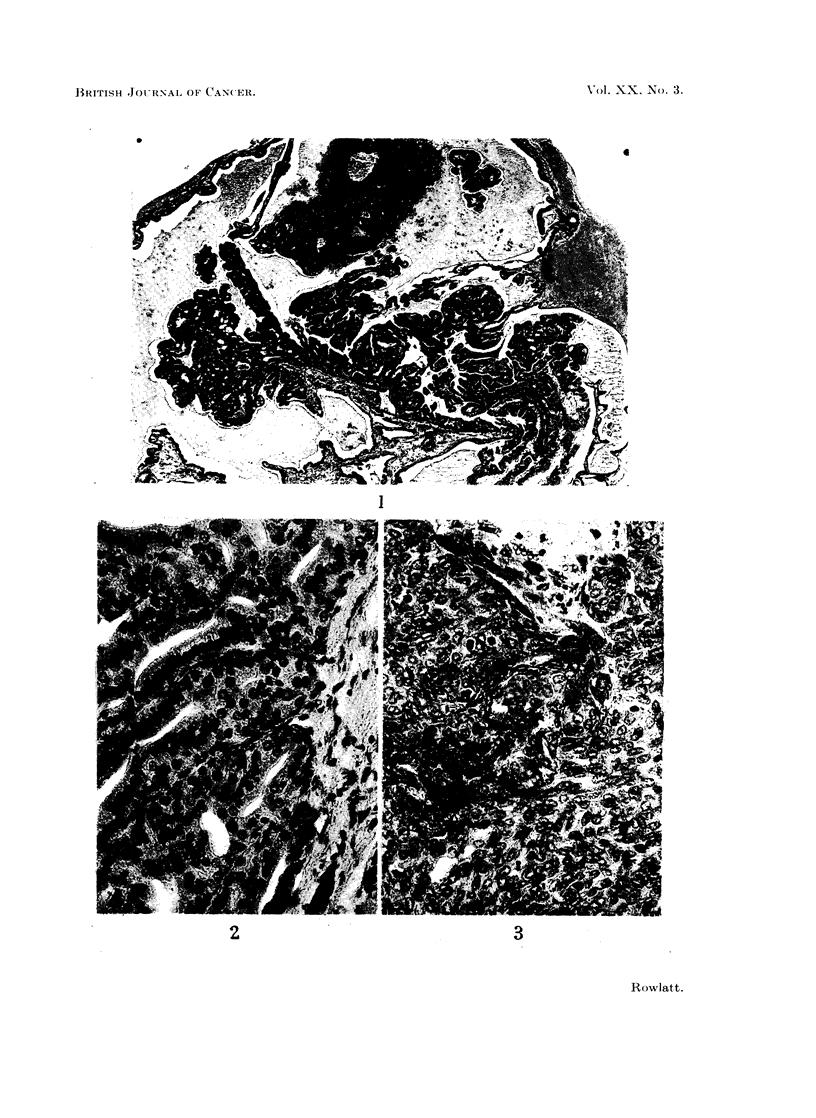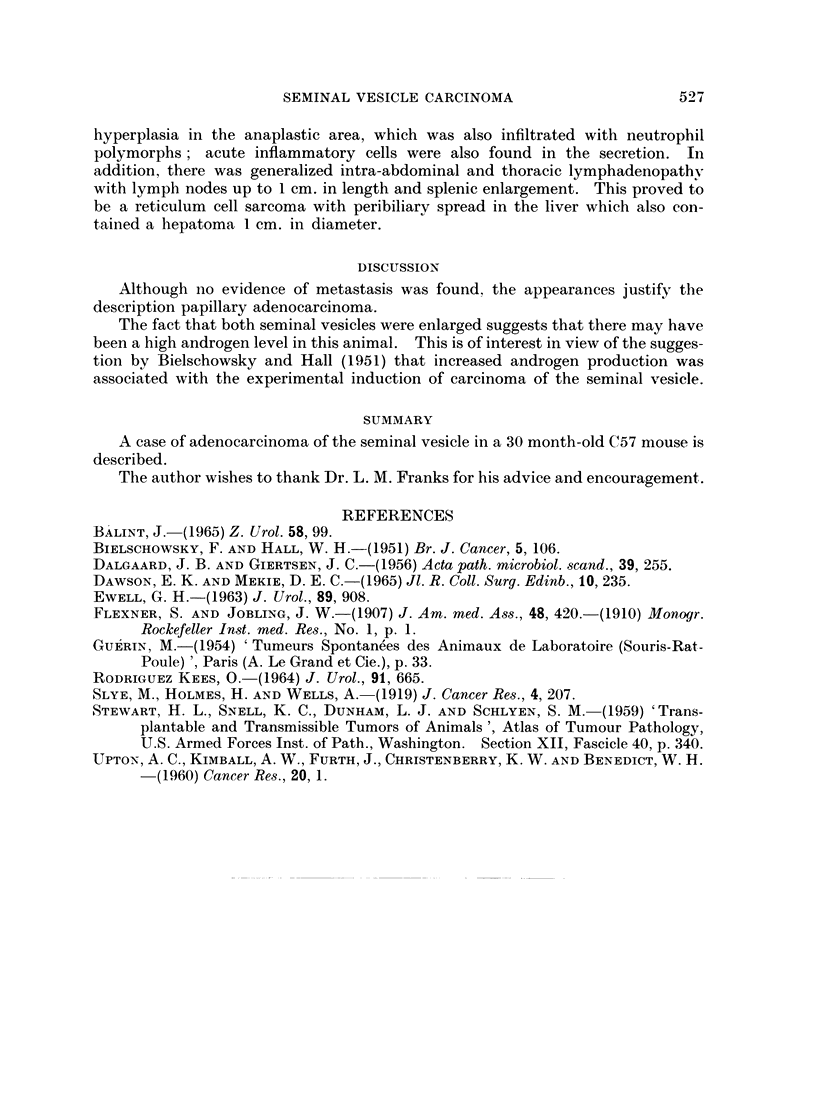# Carcinoma of the seminal vesicle in the mouse.

**DOI:** 10.1038/bjc.1966.64

**Published:** 1966-09

**Authors:** C. Rowlatt

## Abstract

**Images:**


### CARCINOMA OF THE SEMINAL VESICLE IN THE MOUSE

C. ROWLATT

*From the Tissue and Organ Culture Unit, Imperial Cancer Research Fund, London, W.C.2.*

Received for publication May 5, 1966

PRIMARY carcinoma of the seminal vesicle is very rare. Dalgaard and Giertsen (1956) in a critical assessment accepted 23 out of a total of 34 published cases in man and there have been 5 cases reported since (Ewell, 1963 (2 cases); Rodriguez Kees, 1964; Bálint, 1965; Dawson and Mekie, 1965).

Spontaneous malignant tumours of the seminal vesicle are also rare in rodents, although carcinoma of the seminal vesicle has been induced experimentally in rats (Bielschowsky and Hall, 1951). The Flexner-Jobling tumour was originally described as a mixed cell sarcoma (Flexner and Jobling, 1907), later as a teratoma from which an adenocarcinoma developed (Flexner and Jobling, 1910) and now is considered to be a carcinosarcoma (Stewart *et al.*, 1959). A papillary adenoma was found (Upton *et al.*, 1960) in a mouse 19 months after irradiation during the atomic-bomb tests at Eniwetok and 2 spontaneous sarcomas have been reported (Slye, Holmes and Wells, 1919; Guérin, 1954).

In the present paper a carcinoma of seminal vesicle in a mouse is described.

### MATERIALS AND METHODS

The tumour was found in an apparently healthy C57Bl male mouse killed at 30 months. Tissue for light microscopy was fixed in Zenker's fluid and paraffin sections were stained with haematoxylin and eosin.

### FINDINGS

To the naked eye the prostate appeared normal but both seminal vesicles were enlarged to approximately 4 cm. in length and about 0·5 cm. in their greatest diameter. The left seminal vesicle contained normal white opalescent rather viscous secretion while in the right the secretion was pale fawn in colour although of normal consistency. On section there was a papillary tumour in the right seminal vesicle (Fig. 1). The structure was varied and included relatively well differentiated papillary (Fig. 1) and small acinar areas (Fig. 2). Another area was anaplastic and the pattern could be described as pseudosarcomatous (Fig. 3). Mitoses were frequent in this area. Dilated blood vessels were found throughout the tumour, and there were areas of degeneration. There was some endothelial

---

EXPLANATION OF PLATE

FIG. 1.—General view of the papillary adenocarcinoma projecting into the lumen of the seminal vesicle. H. & E. 33 ×.

FIG. 2.—A relatively well differentiated area. H. & E. × 330.

FIG. 3.—A more anaplastic area showing the pseudo-sarcomatous pattern. H. & E. × 330.

1

2

3

Rowlatt.

hyperplasia in the anaplastic area, which was also infiltrated with neutrophil polymorphs ; acute inflammatory cells were also found in the secretion. In addition, there was generalized intra-abdominal and thoracic lymphadenopathy with lymph nodes up to 1 cm. in length and splenic enlargement. This proved to be a reticulum cell sarcoma with peribiliary spread in the liver which also contained a hepatoma 1 cm. in diameter.

### DISCUSSION

Although no evidence of metastasis was found, the appearances justify the description papillary adenocarcinoma.

The fact that both seminal vesicles were enlarged suggests that there may have been a high androgen level in this animal. This is of interest in view of the suggestion by Bielschowsky and Hall (1951) that increased androgen production was associated with the experimental induction of carcinoma of the seminal vesicle.

### SUMMARY

A case of adenocarcinoma of the seminal vesicle in a 30 month-old C57 mouse is described.

The author wishes to thank Dr. L. M. Franks for his advice and encouragement.

### REFERENCES

BALINT, J.—(1965) *Z. Urol.* **58**, 99.
BIELSCHOWSKY, F. AND HALL, W. H.—(1951) *Br. J. Cancer*, **5**, 106.
DALGAARD, J. B. AND GIERTSEN, J. C.—(1956) *Acta path. microbiol. scand.*, **39**, 255.
DAWSON, E. K. AND MEKIE, D. E. C.—(1965) *Jl. R. Coll. Surg. Edinb.*, **10**, 235.
EWELL, G. H.—(1963) *J. Urol.*, **89**, 908.
FLEXNER, S. AND JOBLING, J. W.—(1907) *J. Am. med. Ass.*, **48**, 420.—(1910) *Monogr. Rockefeller Inst. med. Res.*, No. 1, p. 1.
GUÉRIN, M.—(1954) ' Tumeurs Spontanées des Animaux de Laboratoire (Souris-Rat-Poule) ', Paris (A. Le Grand et Cie.), p. 33.
RODRIGUEZ KEES, O.—(1964) *J. Urol.*, **91**, 665.
SLYE, M., HOLMES, H. AND WELLS, A.—(1919) *J. Cancer Res.*, **4**, 207.
STEWART, H. L., SNELL, K. C., DUNHAM, L. J. AND SCHLYEN, S. M.—(1959) 'Transplantable and Transmissible Tumors of Animals ', Atlas of Tumour Pathology, U.S. Armed Forces Inst. of Path., Washington. Section XII, Fascicle 40, p. 340.
UPTON, A. C., KIMBALL, A. W., FURTH, J., CHRISTENBERRY, K. W. AND BENEDICT, W. H. —(1960) *Cancer Res.*, **20**, 1.